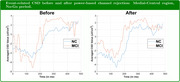# A power‐sensitive approach to preserving regional specificity in EEG signal preprocessing: with applications to localized brain activity analysis for NC and MCI

**DOI:** 10.1002/alz70856_104104

**Published:** 2025-12-24

**Authors:** Ming Gu, Boxin Sun, Tim Martin, Voyko Kavcic, Bruno Giordani, Tongtong Li

**Affiliations:** ^1^ Michigan Alzheimer's Disease Research Center, Ann Arbor, MI, USA; ^2^ Michigan State University, East Lansing, MI, USA; ^3^ Kennesaw State University, Kennesaw, GA, USA; ^4^ Wayne State University, Detroit, MI, USA; ^5^ International Institute of Applied Gerontology, Ljubljana, Slovenia; ^6^ University of Michigan, Ann Arbor, MI, USA

## Abstract

**Background:**

Traditional EEG preprocessing approaches generally reconstruct signals in contaminated channels using interpolation by averaging the signals in neighboring channels. This further reduces the spatial resolution of the EEG signals and raises challenges in localized brain activity analysis, especially in task‐EEG. To overcome this limitation, we propose a power‐sensitive channel rejection (PSCR) approach allowing EEG signals to be processed without sacrificing regional specificity.

**Method:**

Our research focused on task‐based EEG (64‐channel) acquired at Wayne State University (WSU), where participants with subjective cognitive complaints were asked to perform a motion direction discrimination task. The current dataset includes 82 consensus‐diagnosed, community‐dwelling African Americans (ages 60‐90 years, 50 normal cognition (NC) and 32 mild cognitive impairment (MCI)) recruited through WSU and Michigan Alzheimer's Disease Research Center. Each task trial is divided into the following time periods: Stimulus Onset, Go‐indication to Motion‐Stop, NoGo‐indication to Motion‐Stop, Button‐Press period.

Artifacts in EEG signals often appear as segments with abnormal and abrupt changes in amplitude in individual channels. This will be reflected as segments with exceptionally high‐power levels in the current source density (CSD). To reduce the noise effect, signals from adjacent channels are often averaged to formulate a region‐of‐interest (ROI). For each task period, to preserve regional specificity in localized brain activity analysis, we propose a power sensitive channel rejection approach, where a threshold is set based on statistical analysis (with 95% of confidence) and all the channels with CSD power level exceeding the threshold are excluded when calculating the ROI signal.

**Result:**

We compared event‐related regional‐level CSD before and after power‐based channel rejection. It was observed that when the proposed PSCR approach is applied, the differences in localized ROI neural activity between NC and MCI become more distinct. For instance, during the NoGo period, MCI exhibited noticeably higher neural activity in the medial‐central region, possibly compensating for weakened activity in other regions.

**Conclusion:**

The proposed PSCR approach minimizes the interference between the ROIs and helps distinguish the differences in neural activity between NC and MCI. Potentially, this approach may benefit a wide spectrum of EEG‐based brain research.

**Funding**: NSF‐2032709/Li; NIH‐1R21AG046637‐01A1/Kavcic and NIH‐1R01AG054484‐01A1/Kavcic; NIH‐P30AG072931/Paulson; NIH‐P30AG024824/Yung.